# Parathyroid Hormone Related-Protein Promotes Epithelial-to-Mesenchymal Transition in Prostate Cancer

**DOI:** 10.1371/journal.pone.0085803

**Published:** 2014-01-22

**Authors:** Weg M. Ongkeko, Doug Burton, Alan Kiang, Eric Abhold, Selena Z. Kuo, Elham Rahimy, Meng Yang, Robert M. Hoffman, Jessica Wang-Rodriguez, Leonard J. Deftos

**Affiliations:** 1 Division of Otolaryngology-Head and Neck Surgery, Department of Surgery, University of California San Diego, La Jolla, California, United States of America; 2 Department of Medicine, Veterans Administration San Diego Healthcare System, University of California San Diego, La Jolla, California, United States of America; 3 AntiCancer, Inc., San Diego, California, United States of America; 4 Department of Pathology, University of California San Diego and the Veterans Administration San Diego Healthcare System, San Diego, California, United States of America; University of South Alabama Mitchell Cancer Institute, United States of America

## Abstract

Parathyroid hormone-related protein (PTHrP) possesses a variety of physiological and developmental functions and is also known to facilitate the progression of many common cancers, notably their skeletal invasion, primarily by increasing bone resorption. The purpose of this study was to determine whether PTHrP could promote epithelial-to-mesenchymal transition (EMT), a process implicated in cancer stem cells that is critically involved in cancer invasion and metastasis. EMT was observed in DU 145 prostate cancer cells stably overexpressing either the 1-141 or 1-173 isoform of PTHrP, where there was upregulation of Snail and vimentin and downregulation of E-cadherin relative to parental DU 145. By contrast, the opposite effect was observed in PC-3 prostate cancer cells where high levels of PTHrP were knocked-down via lentiviral siRNA transduction. Increased tumor progression was observed in PTHrP-overexpressing DU 145 cells while decreased progression was observed in PTHrP-knockdown PC-3 cells. PTHrP-overexpressing DU 145 formed larger tumors when implanted orthoptopically into nude mice and in one case resulted in spinal metastasis, an effect not observed among mice injected with parental DU 145 cells. PTHrP-overexpressing DU 145 cells also caused significant bone destruction when injected into the tibiae of nude mice, while parental DU 145 cells caused little to no destruction of bone. Together, these results suggest that PTHrP may work through EMT to promote an aggressive and metastatic phenotype in prostate cancer, a pathway of importance in cancer stem cells. Thus, continued efforts to elucidate the pathways involved in PTHrP-induced EMT as well as to develop ways to specifically target PTHrP signaling may lead to more effective therapies for prostate cancer.

## Introduction

Parathyroid hormone-related protein (PTHrP) possesses a variety of physiological and developmental functions but is also known to facilitate the progression of many cancers including prostate cancer. We and others have previously shown that PTHrP stimulates prostate cancer cell growth, invasion and metastasis, operating via both paracrine/autocrine and intracrine pathways [1]–[3]. PTHrP is known to activate a variety of mitogenic pathways including MAPK and PI3K/Akt as well as pathways that stimulate skeletal metastases, one of the most common life-threatening disorders associated with cancer [4]–[6].Secreted PTHrP is known to mediate its cellular effects via interaction with the G-protein-coupled PTH/PTHrP receptor [7]. Co-expression of PTHrP and its receptor has previously been identified in prostate cancer primary tumors and their corresponding bone metastases [8]. Additionally, Freemont et al have previously reported an increase in expression of PTHrP receptor in prostate cancer bone metastases compared to primary tumors, suggesting a potential role of the receptor-mediated pathway in the formation of skeletal metastases[9].

Epithelial-to-mesenchymal transition (EMT) is a process in which epithelial cells undergo cytoskeletal and morphological changes to acquire a mesenchymal phenotype and is important in normal processes such as fibrosis [10]. Due to its effects on cell adhesion and mobility EMT is also critically involved in cancer metastasis and invasion [11], [12]. EMT may be characterized by loss of epithelial markers such as E-cadherin and increased expression of mesenchymal proteins including vimentin and N-cadherin [13]. The transcription factors Snail, Slug and Twist are known to repress E-cadherin expression and induce EMT [14]–[16]. Other oncogenic pathways including Src, Ras, Wnt/β-catenin, PI3K/Akt, MAPK, and TGF-β have all been linked to EMT [17]. Multiple studies have shown that cancer cells become more invasive and metastatic after undergoing EMT. In addition, EMT has been shown to confer stem cell properties to breast cancer cells [18].

Given that PTHrP has a role in promoting invasion and metastasis in prostate cancer and that EMT is one of the main regulators of these properties in cancer, the crucial question presented is whether PTHrP is capable of promoting EMT in cancer cells. PTHrP has been shown to induce EMT in a few contexts, including during parietal endoderm formation and renal fibrogenesis [19], [20], although the ability of PTHrP to regulate EMT in cancer has remained uninvestigated. In breast cancer, the pro-metastatic effects of TGF-β, a potent inducer of EMT, has been shown to be mediated by PTHrP [21]. Taken together, the existing literature suggests that regulation of EMT by PTHrP in cancer is highly likely. In this study we sought to determine the role of PTHrP in regulating EMT in prostate cancer cells along with invasion and metastasis. Establishing a role of PTHrP in regulating bone metastasis cancer and EMT provides both basic and clinical rationales for elucidating the molecular mechanism of PTHrP's actions in many common cancers like prostate.

## Materials and Methods

### Ethics Statement

The VA IACUC approved this study. All animal experiments were performed in accordance with the Guidelines for the Care and Use of Laboratory Animals (NIH Publication Number 85-23) under assurance number A3873-01. The animals were kept under isoflurane anesthesia during surgery, and all efforts were made to minimize suffering. These animals were closely observed, checked daily and euthanized at the first signs of discomfort. Signs of discomfort include any abnormal movements, abnormal feeding or drinking behaviors, lack of self-grooming or any other abnormal behaviors.

### Cells

The DU 145 and PC-3 human prostate cancer cell lines and were obtained from American Type Culture Collection (Manassas, VA) and grown in monolayer in RPMI 1640 media (MediaTech, Herndon, VA) supplemented with 10% fetal bovine serum (Gemini Bio Products, Woodland, CA) at 37°C in a humidified incubator with 95% air, 5% CO_2_. The DU 145 cell line was selected because it has a low constitutive PTHrP expression and does not grow or metastasize well in mouse tumor models, in contrast to PC-3 cells [22]–[24]. The PC-3 cell line, which was originally isolated from a prostate adenocarcinoma that had metastasized to the bone, has an osteolytic phenotype in the immunocompromised mouse models [22], [25].

### Plasmid construction

The PTHrP expression plasmids used in this study were human prepro- PTHrP1–87, PTHrP1-141, and PTHrP1–173; the prepro forms were used to facilitate PTHrP secretion. The constructs were directionally subcloned in the pCI-neo expression vector (Promega, Madison, WI) and the fidelities of all plasmids were confirmed by DNA sequencing and site-specific PTHrP immunoassays [23], [26].

### PTHrP transfection and lentiviral-mediated silencing

The DU 145 prostate cells were seeded at a density of 2×10^4^ cells/cm^2^ in 12-well cell culture dishes and transfected with 1 µg plasmid per well. Individual G418 resistant colonies (800 µg G418/ml) were isolated 21–30 days later. The conditioned media from the picked cell colonies were evaluated for PTHrP expression by site-specific immunoassays and the PTHrP expressing stable DU145 cells were expanded [23], [26]. It has previously been shown that wild type and vector-transfected DU 145 exhibit a similar phenotype [27], [28]. Wild type parental DU 145 cells were thus used as a control for RT-qPCR and matrigel invasion experiments, however parallel experiments were performed with an empty vector-transfected control derivative. The PC-3 prostate cells underwent a stable knockdown of PTHrP via lentiviral siRNA. Control cells were subjected to lentiviral siRNA transduction with a non-targeting sequence construct.

### Quantitative Reverse-Transcription PCR

Cells were harvested two days after being passaged at about 70–80% confluence. Total cell lysate was collected and RNA was extracted using an RNeasy kit (Qiagen). cDNA was synthesized using Superscript III Reverse Transcriptase (Invitrogen, Carlsbad, CA) according to the manufacturer's instructions. Real-time PCR reaction mixes were prepared using Power SYBR Green (Applied Biosystems, Foster City, CA), and run on the 7300 Real-time PCR System (Applied Biosystems) using the following program: 95°C for 10 min, 95°C for 30 s, and 60°C for 1 min, for 40 cycles. Results were analyzed using the Comparative ddCt method and melting curves were performed to ensure product specificity. Experiments were done in technical triplicates and were repeated at least twice independently. GAPDH gene expression was measured as endogenous control. Primers were custom ordered (Eurofins MWG Operon, Huntsville, AL) using the following sequences:

Snail Forward 5′-TCTGAGTGGGTCTGGAGGTG-3′, Snail Reverse 5′- CTCTAGGCCCTGGCTGCTAC-3′, GAPDH Forward 5′-CTTCGCTCTCTGCTCCTCC -3′, GAPDH Reverse 5′-CAATACGACCAAATCCGTTG -3′, E-cadherin Forward 5′-GGCGGAGAAGAGGACCAGGACT-3′, E-cadherin Reverse 5′-TGGCAGGGCGGGGAAGATACC-3′, Vimentin Forward 5′- GGAAATGGCTCGTCACCTTCGT-3′, Vimentin Reverse 5′-AGAAATCCTGCTCTCCTCGCCT-3′.

### Immunofluorescence

Cells were trypsinized and cultured on cover slips. The cells were fixed with 4% paraformaldehyde and blocked in goat serum in Dulbecco's phosphate buffered saline at room temperature prior to incubation with mouse monoclonal to anti-human vimentin (Sigma Aldrich, St. Louis, MO). Cells were then incubated with a goat anti-mouse FITC conjugated secondary antibody (Chemicon, Temecula, CA) and counterstained with DAPI. Finally, SlowFade Gold antifade reagent (Invitrogen, Carlsbad, CA) was used to mount the cover slips onto slides. Fluorescent images were obtained at 40X using Leica DMIRE2 inverted fluorescence microscope and computer program Simple PCI was used for image capture.

### Matrigel Invasion Assay

Invasion of PC3 and DU 145 cells was measured using a Matrigel invasion assay (Becton Dickinson, Bedford, MA). Transwell inserts of 8 µm pore size were coated with a final concentration of 1 mg/mL of Matrigel in cold serum-free DMEM. Cells were trypsinized, and 500 mL of cell suspension (1×10^5^ cells/mL) were added in triplicate wells. The lower chamber of the transwell was filled with 750 µl of culture media containing 0.5% serum as a chemoattractant and allowed to incubate at 37°C for 48 hours. Invading cells on the lower surface that passed through the filter were fixed and stained using crystal violet in gluteraldehyde and photographed. The number of the stained nuclei was counted in a predetermined and consistent section of each well.

### Animals

Six-month-old male, severe combined immunodeficiency (SCID) mice were housed in a barrier filter room and fed Purina rodent chow ad lib. The animals were bled at the end of the study using a retro-orbital method.

### Orthotopic Tumor Implantation

Subconfluent DU145 and stably transfected DU145-PTHrP(1-173)-GFP cells were freshly trypsinized, counted, and placed on ice immediately before injection. The mice were injected with 10^6^ cells subcutaneous space of the flank of the animal in a total volume of 0.25 ml serum free RPMI 1640 media. The mice were sacrificed to harvest tumor tissue 4 weeks after tumor cell injections. Tumor fragments (1 mm^3^) were freshly prepared from the subcutaneous tumors and implanted into the prostate lateral lobe of another SCID mouse (22–24). The 1 mm^3^ tumor fragments were dissected, measured with calipers, and weighed to assure the exact amount of starting tumor was used for each animal. The prostate capsule was exposed following a lower mid-line abdominal incision and a tumor fragment was inserted into the capsule. The prostate capsule was then closed with an 8-0 surgical suture and the incision in the abdominal wall was closed with a 6-0 surgical suture in one layer [29].

The animals were kept under isoflurane anesthesia during surgery. All procedures of the operation described above were performed with a 7 X magnification microscope. The mice were evaluated 60 days after the tumor implantation for tumor progression and metastasis by fluorometry and for skeletal abnormalities by X-ray. High-magnification imaging of the GFP-expressing tumors was carried out with a Leica fluorescent stereomicroscope, model LZ12, equipped with a 50 W mercury lamp and whole-body imaging was carried out in a light box illuminated by blue light fiber optics (Lightools Research, Encinitas, CA) and imaged using a thermoelectrically cooled color CCD camera (Hamamatsu Photonics, Bridgewater, NJ).

### Intraosseous Injections

To evaluate the effect of PTHrP on prostate cancer growth in bone, we used an intra-tibial model for prostate cancer bone metastasis (1, 24). We studied five types of stably transfected GFP expressing DU145 cells: (1) wild-type, (2) vector (pCI-neo), (3) PTHrP(1–87), (4) PTHrP(1–141) and (5) PTHrP(1–173) transformed cells. The mice were injected with 10^6^ cells in 15 µl sterile PBS into the bone marrow of the right proximal tibia using a 26-gauge needle and a Hamilton glass syringe. The left tibia served as the negative control. The mice were evaluated 60 days after the intraosseous injections for skeletal abnormalities by X-ray [24]. Skeletal X-rays were exposed with 40 keV for 20 seconds in a Faxitron 5000 series X-ray cabinet and Kodak X-Omat TL films were processed in a Kodak film processor. For higher resolution imaging of the bone abnormalities, we used a GE eXplore Micro CT (GE Healthcare).

### PTHrP Immunoassay

Cell extracts and media PTHrP were measured by RIA based on PTHrP(1–34), PTHrP(38–64), and PTHrP(109–141), as described previously [30]. All of the samples were assayed in multiple dilutions and in triplicates.

### Statistical Analysis

All experiments were performed in triplicate and error bars represent standard deviation. Statistical significance was tested using a two sample independent t-test (2-tailed test) with the threshold set at *P*<0.05.

## Results

### PTHrP Overexpression Promotes the Expression of Mesenchymal Markers

**Figure 1:**
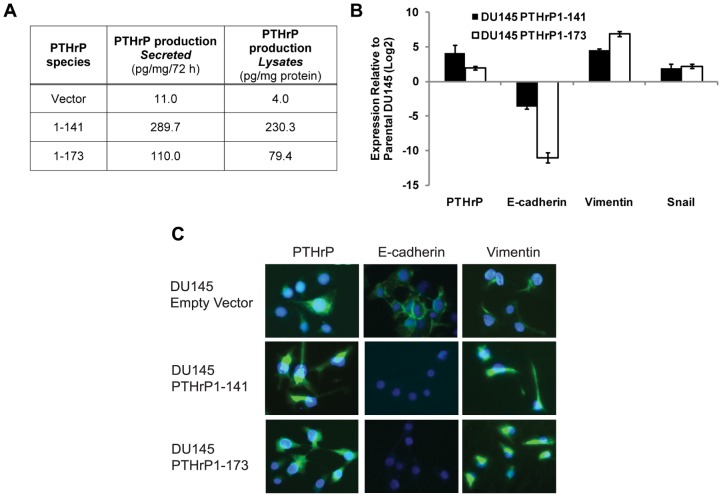
PTHrP overexpression induces EMT. A) Quantification of PTHrP protein levels in control and PTHrP-overexpressing DU 145 cells by immunoassay. B) mRNA expression of PTHrP and EMT markers in control and PTHrP-overexpressing DU 145 cells. C) Immunofluorescence images confirming the overexpression of PTHrP, the upregulation of vimentin, and downregulation of E-cadherin in PTHrP-overexpressing DU 145 cells.

To study regulation of EMT, PTHrP was stably overexpressed in DU 145, a prostate cancer cell line with low basal PTHrP expression as determined by immunoassay (Figure 1A). Two clones were created, with one overexpressing the 1–141 isoform of PTHrP and the other overexpressing the 1–173 isoform (Figure 1B). Both clones displayed markers of EMT, expressing notably higher levels of the mesenchymal proteins snail and vimentin and lower levels of E-cadherin relative to parental DU 145, as determined by qRT-PCR (Figure 1B). Upon stable overexpression of full-length PTHrP-(1–173) in DU 145, vimentin and snail mRNA expression were elevated by 115 and 6.82 fold, respectively, while E-cadherin expression decreased demonstrably by 2080 fold. Similarly, stable overexpression of PTHrP-(1–141) increased vimentin and snail mRNA expression by 23.5 and 3.88 fold, respectively, and decreased E-cadherin expression by 12.3 fold. Data is shown with the wild-type parental derivative as a control. In a parallel experiment, PTHrP-overexpressing DU 145 cells demonstrated a 6.06 fold induction of snail and 9.68 decrease in E-cadherin expression as compared to the empty vector-transfected DU 145 derivative (data not shown). The induction of EMT is verified by an immunofluorescence assay. Overexpressions of both isoforms of PTHrP demonstrate a decrease in E-cadherin with an increase in vimentin protein expression (Figure 1C).

### PTHrP Knockdown Reduces Expression of Mesenchymal Markers

**Figure 2:**
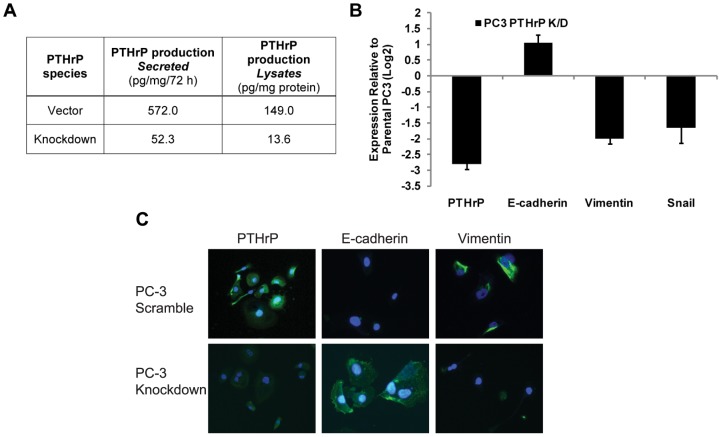
PTHrP Knockdown reverses EMT. A) Quantification of PTHrP protein levels in control and PTHrP-knockdown PC-3 cells by immunoassay. B) mRNA expression of PTHrP and EMT markers in control and PTHrP-knockdown PC-3 cells. C) Immunofluorescence images confirming the overexpression of PTHrP the upregulation of vimentin, and downregulation of E-cadherin in control and PTHrP-knockdown PC-3 cells.

To further demonstrate the regulation of EMT by PTHrP in prostate cancer, permanent PTHrP knockdown via retroviral transduction was performed in PC-3, a prostate cancer cell line with high basal PTHrP expression as determined by immunoassay (Figure 2A). In PC-3-KD cells, expression of PTHrP was reduced to 15% of the control PC-3 (Figure 2B), while levels of Snail and vimentin were down-regulated by 3.03 and 3.73 fold, respectively, and E-cadherin expression was elevated by 2.30 fold (Figure 2B). The change in EMT protein expression is demonstrated with an immunofluorescence assay. Knocking down PTHrP downregulates vimentin and upregulates E-cadherin, which confirms the qPCR data (Figure 2C). Together with Figure 1, these data suggest that PTHrP activity or expression promotes EMT in prostate cancer.

### PTHrP Regulates Invasion and Upregulation of MMP-9

**Figure 3:**
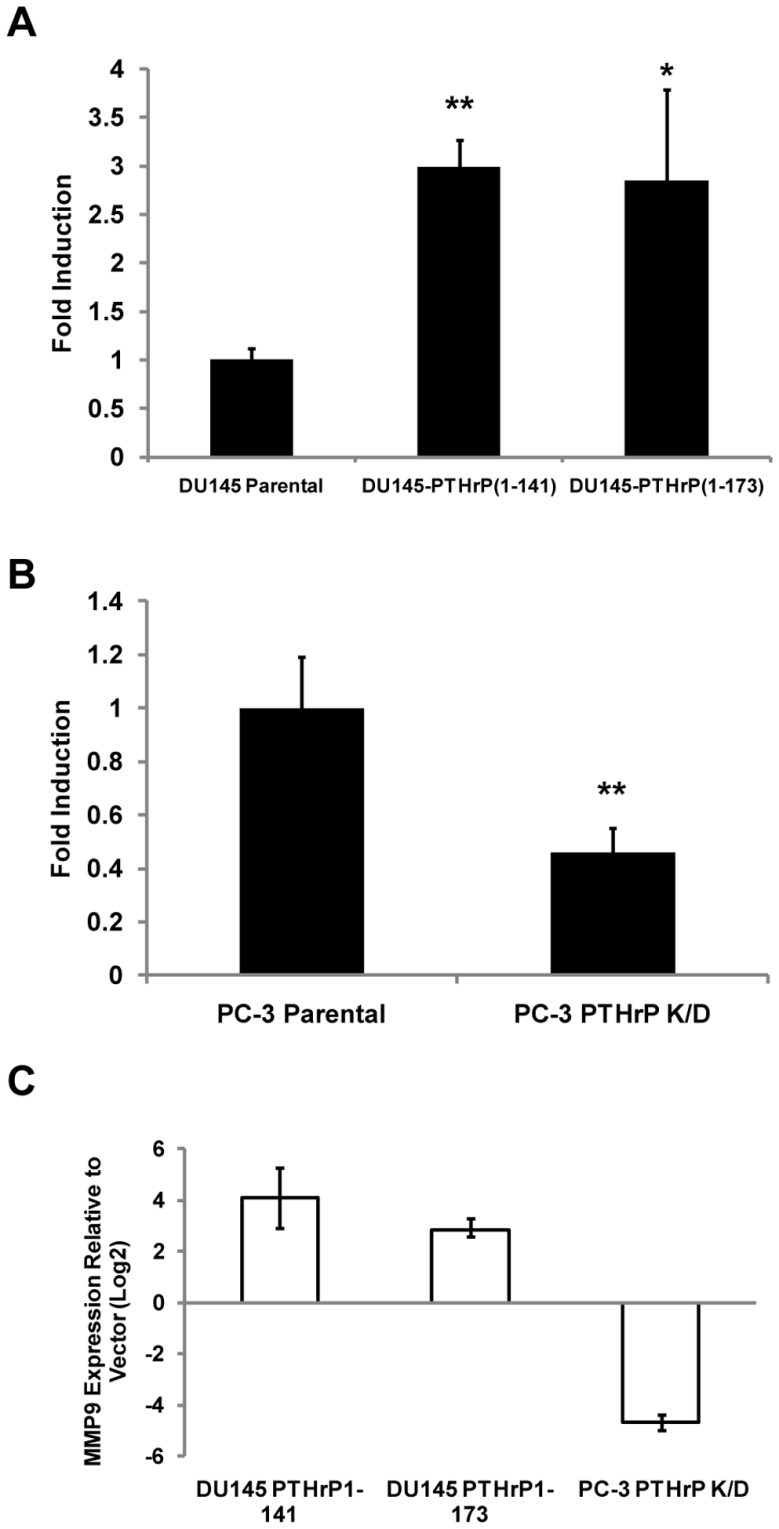
PTHrP Promotes Invasion and MMP-9 Expression. A) Matrigel invasion assay showing increase in invasion of DU145 cells upon overexpression of either PTHrP 1–141 or 1–173. B) Matrigel invasion assay showing decrease in invasion of PC3 cells upon knockdown of PTHrP. C) mRNA levels of MMP-9 in PTHrP-overexpressing DU145 cells and PTHrP-knockdown PC3 cells relative to their respective controls. * indicates p<.05, ** indicates p<.01.

In order to establish that activation of PTHrP or EMT results in increased invasiveness in our experimental system, a matrigel invasion assay was performed to determine the relative invasiveness of DU 145-PTHrP(1–141), DU 145-PTHrP(1–173), and PC-3-KD cells compared to their respective controls. DU 145-PTHrP(1–141) and DU 145-PTHrP(1–173) cells were found to be 3.0 (p<.01) and 2.9 (p<.05) times more invasive than parental DU 145 cells, respectively (Figure 3A). Meanwhile, PC-3-KD cells were found to be only 0.5 times (p<.01) as invasive as parental PC-3 cells (Figure 3B). In a parallel experiment, PTHrP-(1–141) and -(1–173) DU 145 cells demonstrated a 2.0 (p<0.5) and 2.1 fold (p<.05) increase in matrigel invasion as compared to the empty vector-transfected DU 145 derivative, respectively (data not shown). Lastly, PTHrP overexpression in DU 145 was observed to upregulate the expression of MMP-9 by 17.1 and 7.10 fold in PTHrP-(1–141) and PTHrP-(1–173) expressing derivatives, respectively, while knockdown in PC-3 downregulated MMP-9 by 25.1 fold (Figure 3C).

### PTHrP Promotes Tumor Growth and Bone Destruction in an Orthotopic/Intraosseous Mouse Model

**Figure 4:**
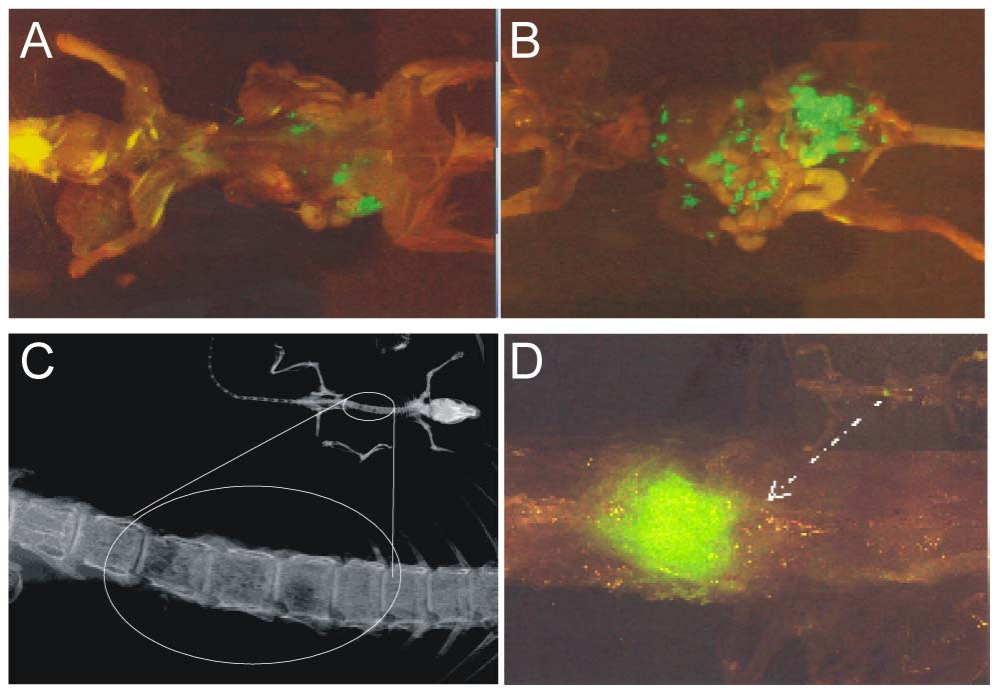
PTHrP Promotes Tumorigenesis in Orthotopic Mouse Model. A) Orthotopic mouse model showing a mouse injected with DU145-GFP cells. B) Mouse injected with DU 145-PTHrP(1–173) cells. C) Evidence of bone destruction in mouse injected with DU 145-PTHrP(1–173) cells. D) Spine metastasis in a mouse injected with DU 145-PTHrP(1–173) cells.

**Figure 5:**
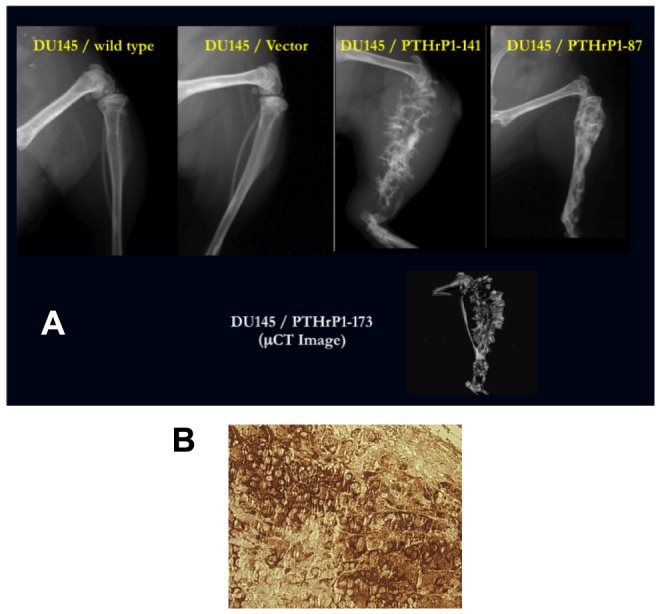
PTHrP Promotes Destruction of Bone in an Intraosseous Model. A) X-ray and uCT Images showing the effect of PTHrP overexpression on bone destruction in an intraosseous mouse model. Tibia of mice receiving PTHrP-overexpressing DU 145 displayed significant destruction of bone compared to control. B) The DU 145–GFP–PTHrP1-173 prostate tumor was removed from the mouse, fixed and stained immunohistochemically for PTHrP using a biotin-streptavidin- horseradish peroxidase system (brown reaction product indicates PTHrP expression).

DU 145 and DU 145-PTHrP(1–173) cells were stably transfected with a GFP expression plasmid and implanted orthotopically into the prostate bed of nude mice or directly into bone, as previously described (1). Although all mice formed tumors, those implanted with DU 145-PTHrP(1–173) cells formed significantly larger tumors compared to those injected with normal DU 145 cells. Representative images show extensive tumor mass in mice injected with DU 145-PTHrP(1–173) cells compared to mice injected with parental DU 145 (Figure 4A). In addition, metastasis to the spine was observed in one of the mice injected with DU 145-PTHrP(1–173), while no mice injected with DU 145 formed metastases (Figure 4B). DU 145 or DU 145-vector cells formed no tumor when injected into mice tibiae while DU 145-PTHrP(1–141) or DU 145-PTHrP(1–173) cells injected into mice tibiae resulted in tumor formation, invasion and destruction of bone (Figure 5), confirming the well established role of PTHrP in bone resorption. Lastly, expression of PTHrP in tumors were confirmed by immunohistochemistry (Figure 5B).

## Discussion

We have identified PTHrP not only as a critical mediator of tumor progression in prostate cancer, but also as a promoter of EMT. While PTHrP has been shown to induce EMT in development [10], [31], our observation that it promotes EMT in cancer as well remains a novel finding. This finding implies that PTHrP may have an even more significant role in cancer progression than previously believed, since the ability to regulate EMT implies the potential to regulate a variety of properties related to cancer progression including invasion, metastasis, cell-motility, cell-adhesion, angiogenesis, and stemness/tumorigenicity [11], [18], [32]–[34]. Prognosis for advanced prostate cancer remains poor due to frequent bone metastasis and invasion [35], [36]. Thus, targeting of PTHrP may lead to more effective therapies for prostate cancer.

Our data showed that PTHrP overexpression in DU 145 cells induced EMT and promoted invasion, tumorigenicity, and metastasis, while PTHrP knockdown in PC-3 cells induced contrary effects. Furthermore we observed that the PC-3 cell line has high basal expression of PTHrP as determined by PTHrP immunoassay and is inherently more metastatic and invasive compared to DU 145, which has low basal PTHrP expression. These observations posit that PTHrP may not only facilitate metastasis by promoting bone resorption, but may also be an important regulator of aggressive phenotype in prostate cancer. Both 1–141 and 1–173 isoforms of PTHrP were found to promote EMT, consistent with the fact that the classical active site is located near the amino-terminus as shown by previous studies [37]; however, additional processed peptides of PTHrP may also be important mediators of this action. Targeting all isoforms of PTHrP for anti-cancer therapy may necessary, although targeting of the 1–141 isoform may be of primary importance as it accounts for the majority of PTHrP expression in humans.

Downstream targets that are activated by PTHrP include Snail, AP-1, CREB, ERK1/2, VEGF, PI3K/Akt and Cyclin D1 [20], [38]–[45]. Snail promotes EMT through direct transcriptional repression of E-cadherin [15]. CREB upregulates VEGF which in turn promotes EMT, invasion and angiogenesis [46], [47]. The PI3K/Akt pathway is a key regulator of cell proliferation and has also been shown to induce EMT in a variety of cancers [48]. AP-1 was shown to be involved in TGF-β-induced EMT [49]. Overexpression of cyclin D1 has been shown to induce glioma invasion by increasing metalloproteinase activity and cell motility [50]. Prevailing studies show that PTHrP, TGF-β, EGF, and VEGF cooperate through activation of ERK1/2 to induce EMT during renal fibrogenesis [19], and that Snail is an immediate early target of PTHrP in murine parietal endoderm formation [20]. One or a combination of these pathways is likely to explain the induction of EMT and invasion that were observed in our experiments, in which we confirmed the induction of Snail by PTHrP. Though it has been shown previously that PTHrP is able to upregulate Snail transcription in the absence of de novo protein synthesis [20], it remains unclear whether this effect is through direct binding to the Snail promoter or through activation of signaling pathways such as Akt that are known to regulate Snail. Either way, further studies would be needed to confirm the mechanism of PTHrP-induced EMT in prostate cancer.

It is possible that various other pathways rely on PTHrP to promote EMT, invasion and metastasis. TGF-β, a potent inducer of EMT, has been shown in breast cancer to promote PTHrP expression resulting in bone destruction [21]. Various oncoproteins including Ras, Tpr-Met, Src have all been shown to target PTHrP and also have proven roles in EMT, invasion and metastasis [51]–[53]. Of particular interest would be Indian hedgehog, which is known to regulate PTHrP during early bone and cartilage growth [54], [55]. Members of the hedgehog family are aberrantly activated in a variety of cancers including prostate cancer, have been shown to indirectly promote EMT, and are theorized to have a role in the transformation of adult stem cells into cancer stem cells [56]–[59]. It would be interesting to determine whether Ihh relies on PTHrP for induction of EMT and other malignant properties in cancer.

Ongoing research continues to reinforce the theory that cancer stem cells are the main drivers of cancer progression and key determinants of therapeutic response [60]. Thus, an important question to consider is whether PTHrP may regulate prostate cancer stem cells through an EMT-mediated pathway. As EMT has been shown previously to induce cancer stem cell properties [18], it follows that PTHrP should potentially be able to regulate stem cell properties in prostate cancer and thus may be a valuable therapeutic target for preventing recurrence and metastasis. Prostate cancer stem cells have previously been isolated and characterized by a CD44^+^/CD133^+^/α2β1^hi^ phenotype [61]. Future work must focus on the ability of PTHrP to regulate this compartment in prostate cancer.

The finding that PTHrP induces EMT while promoting invasion and tumor growth suggests that treatments that target PTHrP may be used in conjunction with conventional treatments for inhibiting invasion and metastasis in prostate cancer, especially for recurrent tumors. We have previously screened a library of compounds and identified several that are capable of inhibiting PTHrP expression and cell growth in lung cancer [62]. It would be of great clinical interest to extend these results to prostate cancer and to determine whether such drugs are also capable of blocking PTHrP-induced EMT and invasion. Meanwhile, further efforts to characterize the pathways involved in PTHrP-induced EMT may lead to the elucidation of a role for PTHrP in cancer stem cell development and to novel therapies that could significantly improve the prognosis of metastatic prostate cancer (1, 57).
